# Flavonoid, Nitrate and Glucosinolate Concentrations in *Brassica* Species Are Differentially Affected by Photosynthetically Active Radiation, Phosphate and Phosphite

**DOI:** 10.3389/fpls.2019.00371

**Published:** 2019-03-27

**Authors:** Libia Iris Trejo-Téllez, Elías Estrada-Ortiz, Fernando Carlos Gómez-Merino, Christine Becker, Angelika Krumbein, Dietmar Schwarz

**Affiliations:** ^1^Colegio de Postgraduados, Montecillo, Mexico; ^2^Department of Crop Protection, Hochschule Geisenheim University, Geisenheim, Germany; ^3^Leibniz Institute of Vegetable and Ornamental Crops, Großbeeren, Germany

**Keywords:** Brassicaceae, biostimulation, secondary metabolites, nutraceutics, PAR, phosphorus(deficiency, uptake)

## Abstract

We evaluated the effects of phosphate (Pi-deficiency: 0.1 mM; Pi-sufficiency: 0.5 mM), phosphite (low-Phi: 0.1 mM; medium-Phi: 0.5 mM; and high-Phi: 2.5 mM), and two mean daily photosynthetically active radiations (lower PAR: 22.2 mol ⋅ m^-2^ ⋅ d^-1^; higher PAR: 29.7 mol ⋅ m^-2^ ⋅ d^-1^), as well as their interactions, on flavonoid, nitrate and glucosinolate (GL) concentrations and growth characteristics in hydroponically grown *Brassica campestris* cv. Mibuna Early and *Brassica juncea* cv. Red Giant. As expected, higher PAR increased dry matter and contrariwise decreased number of leaves but only in *B. campestris*. Total flavonoid and individual flavonoid compounds increased with the higher PAR value in *B. campestris*. Pi-sufficiency resulted in a lower quercetin concentration in both species, the isorhamnetin and total flavonoid concentrations in *B. campestris*, and the cyanidin concentration in *B. juncea*, in comparison to Pi-deficiency. Similarly, Pi-sufficient plants exhibited lower GL concentration, especially alkyl-GLs in *B. campestris* and alkenyl-GLs and an aryl-GL in *B. juncea*. Pi did not affect the nitrate concentration in either species, and nor did Phi influence the flavonoid concentrations in either species. In *B. campestris*, medium Phi (0.5 mM) increased the 1-methoxyindol-3-ylmethyl GL concentration by 28.3%, as compared to that observed at low Phi. In *B. juncea*, high Phi level increased the but-3-enyl-GL concentration by 18.9%, in comparison to values recorded at medium Phi. *B. campestris* plants exposed to higher PAR increased total flavonoids concentration. In both *Brassica* species, higher PAR stimulated the alkyl-, alkenyl-, and indole-GLs. The interaction of lower PAR and increasing Phi significantly decreased flavonoid concentration in *B. juncea*, whereas increasing Phi at higher PAR increased such concentration in this species. The same combination reduced the concentration of 2-phenylethyl- and indol-3-ylmethyl-GL in *B. juncea*. The highest indol-3-ylmethyl-GL concentration was observed when Pi was deficient combined with medium Phi in *B. juncea*. Thus, PAR, Pi and Phi may modulate flavonoid, GL and nitrate concentrations in *Brassica* species, which may be a useful tool to improve the nutraceutical quality of these leafy vegetables if properly managed.

## Introduction

Solar radiation is a key environmental signal that regulates most forms of life on Earth. Among the relevant bands along the solar radiation spectrum, the visible one (photosynthetically active radiation, PAR) makes up 43.9% of total solar radiation, energy, and its interaction with other environmental cues, including nutrient supply, may affect primary and secondary metabolism in plants (Fallovo et al., 2009, 2011). Particularly, it is well documented that sulfur and phosphorus (P) deficiencies stimulate the synthesis of secondary metabolites such as flavonoids and glucosinolates (GLs) (Lunde et al., 2008; Pant et al., 2015).

Phosphorus is one of the primary nutrients required by plants, making up about 0.2% of their dry biomass (Herrera-Estrella and López-Arredondo, 2016). This macronutrient is an essential component of biomolecules such as sugar phosphates, phospholipid, phosphoproteins, enzymes, and energy-rich compounds such as ATP and NADP, as well as the nucleic acids DNA and RNA, thus playing a pivotal role in genetic heredity, membrane structure, signal perception and transduction, and metabolism (López-Arredondo et al., 2014; Trejo-Téllez and Gómez-Merino, 2018). The demand for P is supplied by the phosphate form of P (Pi; H_2_PO_4_^-^ or HPO_4_^2-^), which is the sole P-containing nutrient important for optimal plant performance (López-Arredondo et al., 2014). Alternatively, an analog of Pi, phosphite (Phi; H_2_PO_3_^-^ or HPO_3_^2-^) is gaining momentum as a novel biostimulant in agriculture, improving yield and quality of crops, as well as inducing diverse mechanisms of tolerance against stress factors (Gómez-Merino and Trejo-Téllez, 2015, 2016). In the same way as Pi, the Phi molecule displays a tetrahedral structure with formal charge of -3. Nevertheless, instead of having four oxygen (O) atoms distributed evenly at the points of the structure bonded to the P atom located in the center found in Pi, the Phi ion has only three O atoms with a hydrogen (H) atom bonded strongly to the P atom. Hence, Phi is defined an isostere of the Pi anion, in which a H atom replaces one of the O atoms bound to the P atom (Varadarajan et al., 2002; Gómez-Merino and Trejo-Téllez, 2015). The lack of an O atom in Phi and the corresponding charge distribution of the structure significantly changes the nature and reactivity of the resultant molecule. For instance, such changes give Phi increased mobility in plant tissues through both the xylem and the phloem. Such high mobility allows Phi to be absorbed and translocated within the plant more readily than Pi (Ratjen and Gerendas, 2009; Jost et al., 2015). Moreover, Phi-containing salts exhibit a higher solubility than that of their analogous Pi-containing ones, which render Phi uptake by leaf and root a more efficient process (Trejo-Téllez and Gómez-Merino, 2018).

Phi may modify nitrogen (N) metabolism in plants and induce nitrate accumulation in edible tissues (Thao et al., 2009). Nitrate itself is relatively non-toxic but its metabolites may produce adverse physiological effects (Santamaria, 2005). Indeed, Phi is not a proper P-source for plant nutrition, and when applied at high concentrations, it may cause deleterious effects on different physiological processes including photosynthesis (Thao et al., 2008; Zambrosi et al., 2011). Another factor affecting photosynthesis is radiation. For instance, chlorophyll fluorescence decreases with increasing sunlight (Gómez et al., 1998). Furthermore, daily changes in nitrate reductase and carbonic anhydrase activities are antagonistic during the onset of natural radiation (Gómez et al., 1998). Importantly, if Phi is not properly used, it may negatively affect nutrient use efficiency and the whole metabolism (Zambrosi et al., 2011; Ramezani et al., 2017). Conversely, a proper application of Phi may induce positive responses, including an enhanced biosynthesis of secondary metabolites involved in antioxidant responses (Estrada-Ortiz et al., 2013, 2016).

Secondary metabolites produced by plants exhibit enormous structural variation, and consequently display a wide range of biological activities (O’Connor, 2015). Based on their biosynthetic origins, plant secondary metabolites encompass three major groups: (1) flavonoids and allied phenolic and polyphenolic compounds; (2) terpenoids; and (3) N-containing alkaloids and sulfur-containing compounds, including GLs (Crozier et al., 2006). In recent years, flavonoids and GLs have been the focus of much research due to their potential as health-promoting phytochemicals. Flavonoids display antioxidant ability and antimicrobial properties and may reduce the risk of cardiovascular diseases and various types of cancer and chronic diseases (Lee et al., 2007). Among them, the flavonoid quercetin has shown the greatest benefits to human health (Knekt et al., 2002; Williams et al., 2004). Furthermore, flavonoids have a great number of functions in plants. For example, the colorless flavonoids accumulate in the outermost layers of plants, absorbing UV radiation and thus preventing its harmful effects on the internal tissues (Gould and Lister, 2006). Some flavonoids have defense functions against herbivores (Roland et al., 2013), and may modulate the activity of auxin-transporting *P*-glycoproteins as well as that of regulatory proteins such as phosphatases and kinases (Peer and Murphy, 2007).

Flavonoid synthesis can be affected by climate conditions such as temperature and radiation. A reduction in PAR has been associated with low concentrations of flavonoids (Agati and Tattini, 2010; Fallovo et al., 2011). Another factor affecting flavonoids biosynthesis is Pi, since Pi deficiency increases their accumulation (Bariola et al., 1999; Stewart et al., 2001; Misson et al., 2005). Pi deficiency induces important changes in several primary and secondary metabolic pathways. Alteration of secondary metabolism in root tissues under Pi deficiency seems to enhance Pi uptake. Regardless of the physiological mechanism involved, flavonoids biosynthesis is enhanced in nutrient-poor conditions and can help plants to cope with unfavorable environments (Malusà et al., 2006).

Glucosinolates are mainly found in the family Brassicaceae. Some GLs and their breakdown products have attracted intense research because of their cancer-preventing attributes (Plate and Gallaher, 2006; Stoin et al., 2007). The effect of P on the production of GLs such as isothiocyanates is relatively insignificant in mustard (*Sinapis alba* cv. Ida Gold) and radish (*Raphanus sativa* cv. Colonel), and these crops differ significantly in their ability to uptake and accumulate P (Brown et al., 2008). However, P deficiency does increase the total GL concentration in *B. campestris* L. subsp. *chinensis* var. *communis* at normal light intensity, though this effect is not significant with low light intensity (Yang et al., 2009).

Many previous studies have focused on the influence of N and sulfur on GLs biosynthesis (e.g., Schonhof et al., 2007; Fallovo et al., 2011), because these nutrients have a strong impact as amino acid precursors and intermediaries involved in this biosynthetic process. In addition, during the biosynthesis of GLs, there is a high P demand for the formation of phosphorylated cofactors such as uridine diphosphate glucose (UDPG) and co-substrates such as 3′-phosphoadenosine 5′-phosphosulfate (PAPS) (Wittstock and Halkier, 2002). While Pi is the sole source of P important for plant nutrition, Phi is emerging as a novel biostimulant, and may improve some quality attributes in horticultural crops. However, information is missing on the effect of PAR, Pi and Phi and their interactions on the biosynthesis of secondary metabolites such as flavonoids and GLs. Moreover, it is not well understood whether these factors affect the accumulation of nitrate in edible tissues. Hence, the primary aim of this research was to evaluate the main effect of PAR, Pi, Phi, and their interactions, on the concentrations of flavonoids, GLs, and nitrate in two *Brassica* species differing in their concentration and composition of these secondary metabolites (Fallovo et al., 2011). Due to the wide spectrum of health-promoting substances, the two *Brassica* species are important for human consumption.

## Materials and Methods

### Plant Material, Experimental Conditions, and Treatments

Two experiments were conducted in a hydroponic system, under greenhouse conditions (covered with conventional glass), at the Leibniz Institute of Vegetable and Ornamental Crops located in Großbeeren, Germany (13° 20′ east longitude and 52° 22′ north latitude), considering two mean daily PAR levels: the first experiment was carried out from 29 August to 23 September 2013 resulting in 29.7 mol ⋅ m^-2^ ⋅ d^-1^ (higher level) and the second one from 30 September to 30 November 2013 resulting in 22.2 mol ⋅ m^-2^ ⋅ d^-1^ (lower level). All other climate conditions were the same in both experiments, conducted at average temperature of 17.4°C, average relative humidity of 77%, and average CO_2_ concentration of 400 μmol ⋅ mol^-1^. For each experiment, seeds of *B. juncea* cv. Red Giant and *B. campestris* cv. Mibuna Early were germinated in rockwool cubes. Twenty-two days after germination, plants with 2 and 3 true leaves respectively, were transplanted into a recirculating nutrient solution system supported by 7.5-m-long gullies, having 44 plants per gully, being half of each cultivar; spacing between plants was 0.15 m. Eighteen gullies were placed within the greenhouse in a completely randomized experimental design with 12 treatments, each replicated three times.

A factorial experiment, resulting from the treatment combinations of three study factors, namely PAR, Pi, and Phi, was conducted. Pi and Phi were applied in the nutrient solution. The Pi factor was tested at deficiency (0.1 mM) and sufficiency (0.5 mM) levels. Phi was tested at low (0.1 mM), medium (0.5 mM) and high (2.5 mM) levels. Phosphate was obtained from phosphoric acid and the Phi from phosphorous acid, both reagent grade (Carl Roth GmbH, Karlsruhe, Germany). The other nutrients were the same in both experiments and added to the nutrient solution at concentrations (mM) as follows: NO_3_^-^ 7.82, NH_4_^+^ 0.33, K^+^ 3.93, Ca^2+^ 1.95, Mg^2+^ 0.77, SO_4_^2-^ 0.77; the nutrient solution was supplemented with micronutrients at concentrations (μM) as follows: Fe^2+^ 40.0, Mn^3+^ 5.0, Zn^2+^ 4.0, BO_3_^3-^ 30.0, Cu^2+^ 0.5, MoO_4_^2-^ 0.5. Electrical conductivity was kept at 2 dS ⋅ m^-1^ when preparing the nutrient solution with demineralized water in both experiments and changed once a week. The pH was controlled and kept between 5.5 and 6.0.

### Preparation of Samples for Analyses

At commercial maturity (25 and 35 days after transplanting for experiments at 29.7 and 22.2 mol m^-2^ daily mean PAR, respectively), 10 plants were harvested from each treatment and its replications. Commercial maturity for *B. campestris* was defined by the presence of at least 49 leaves and for *B. juncea* of 7 to 8 leaves. For *B. juncea*, a leaf was randomly taken from each of the 10 harvested plants, and then the midrib was cut off and discarded. In *B. campestris*, the petiole was cut off from each of the 10 harvested plants, and then 100-150 g samples were taken in duplicate from each treatment and its replications.

Samples for analysis of flavonoids and GLs were frozen at -20°C in a Poron-brand freezer (Erfurt, Germany). Already-frozen samples were lyophilized in a Christ-brand freeze drier (Martin Christ, Osterode, Germany) for about 1 week. Following this, the samples were finely milled in a Retsch-brand grinder (F. Kurt Retsch GmbH & Co., Haan, Germany) and stored for subsequent analysis.

Samples used for nitrate analysis were dried in a Binder-brand drying oven for 72 h at 70°C, and then finely milled in the same Retsch grinder and stored for subsequent analysis.

### Growth Characteristics and Nitrate Analysis

The number of plant leaves was determined at harvest. After harvest, leaves were dried in a Binder-brand drying oven for 72 h at 70°C to determine leaf dry matter.

Nitrate concentrations were measured potentiometrically in plant tissue extracts with a nitrate ion plus Sure-Flow1 electrode (Orion-Research, Beverly, MA, United States).

### Analyses of Flavonoids

Flavonoids were determined as their aglycones after acid hydrolysis (Fallovo et al., 2011). To do this, 0.25 g of the lyophilized plant material were weighed, and 20 mL of aqueous methanol (62.5%) and 5 mL of HCl (8 M) added. Then it was held at reflux for 2 h in a hot water bath (100°C). After this time, the extract was cooled by immersing it in cold water, and it was adjusted to 50 mL with 50% methanol and sonicated (Bandelin Sonorex RK 100, Berlin, Germany) for 5 min. Subsequently, a sample of the previously homogenized extract was passed through a PTFE filter (0.45 μm, polytetrafluoroethylene; Roth, Karlsruhe, Germany) and placed in a vial for later analysis by HPLC-DAD-ESI-MS (Agilent, Waldbronn, Germany).

The composition and concentration of flavonoids were determined using an 1100 series HPLC (Agilent, Waldbronn, Germany) equipped with a diode array detection system. The extracts were separated on a Prodigy column (ODS 3, 150 × 3.0 mm, 5 μm, 100 Å) (Phenomenex, Aschaffenburg, Germany) with a C18 security guard (ODS 3, 4 × 3.0 mm, 5 μm, 100 Å) at a temperature of 25°C using a water/acetonitrile gradient (Th Geyer GmbH, Renningen, Germany). Solvent A consisted of 99.5% water and 0.5% acetic acid (VWR International, Dresden, Germany), while solvent B was 100% acetonitrile. In this analysis, the following gradient was used: 30–35% B (5 min), 35–39% B (12 min), 39–90% B (5 min), isocratic 90% B (5 min), 90–30% B (5 min), isocratic 30% B (5 min). The injection volume was 50 μL, using a flow rate of 0.3 mL min^-1^, using a wavelength of 370 nm (for quercetin, kaempferol and isorhamnetin) and 520 nm (for cyanidin) for quantification. Dihydroquercetin, kaempferol, isorhamnetin and cyanidin (Carl Roth GmbH, Karlsruhe, Germany) were used as standards for external calibration curves and compound identification based on retention time and characteristic MS signals. The deprotonated molecular ions [M-H]^-^ with m/z 315, 301 and 285 for isorhamnetin, quercetin and kaempferol respectively, and the molecular ion [M]^+^ with m/z 287 for cyanidin were detected by HPLC-DAD-ESI-MS, using Agilent 1100 series MSD equipment, with ESI as a source of ions in negative mode and positive mode, respectively. Nitrogen was used as drying gas (12 L min^-1^, -350°C) and nebulizing gas (40 psi).

### Analyses of Glucosinolates

Desulfo-glucosinolate profiles and concentrations were derived using a modified HPLC protocol (Krumbein et al., 2005). Twenty mg of previously lyophilized plant material were weighed and finely milled, in duplicate, and placed in 2-mL plastic tubes, to which 750 μL of 70% methanol at 70°C were added, shaken for 10 min at 1400 rpm and at 70°C in a DITABIS unit (Model MHL 23, Pforzheim, Germany). Samples were subsequently centrifuged for 5 min at 4500 rpm and at 20°C (Centrifuge Heraeus, D-37520, Osterode, Germany). The resulting supernatants were placed in a plastic tube. The residues were extracted twice more with 500 μL of 70% methanol at 70°C, shaken for 5 min at 1400 rpm and at 70°C; subsequently they were centrifuged for 5 min at 4500 rpm at 20°C, and the supernatants were collected in the same plastic tube used in the previous step. The supernatants were added to a SPE column, which was pre-conditioned with 500 μL of DEAE Sephadex A-25 ion exchanger suspension (Sigma-Aldrich Chemie GmbH, Sweden). Prior to sample loading, the column was first equilibrated in 2 M acetic acid, then pre-treated by the addition of 1 mL aliquots of 6 M imidazole formate (Carl Roth GmbH, Germany) in 30% (v/v) formic acid, followed by two washes with 1 mL deionized water. The column was washed twice with 1 mL 20 mM sodium acetate buffer pH 4.0 (Sigma-Aldrich Chemie GmbH, Germany), and 75 μL purified *Helix pomatia* aryl sulfatase (Roche Diagnostics GmbH, Germany) was loaded and left to stand for 12 h. The desulfo-glucosinolates were eluted from the columns with two applications of 0.5 mL of ultrapure water, then placed in tube filters (Costar Spin-X 0.22 μm of cellulose acetate in 2-mL polypropylene tubes, Corning Incorporated, United States) and centrifuged for 5 min at 3000 rpm at 20 °C for reading. The analysis was performed using a 1290 Infinity HPLC (Agilent Technologies, Germany) with a Poroshell 120 EC-C18 column (2.1 × 100 mm 2.7 Micron, Agilent Technologies, United States) at a temperature of 30°C using a water/acetonitrile gradient (Th. Geyer GmbH, Renningen, Germany). Solvent A consisted of 100% water, and solvent B consisted of 40% acetonitrile in water. The following gradient was used: 99.5% A, 0.5% B (12 min); 50.5% A, 49.5% B (3 min); 0.5% A, 99.5% B (3 min); 99.5% A, 0.5% B (1 min). The injection volume was 5 μL, using a 0.4 mL min^-1^ flow and a 229 nm wavelength for quantification. The GL concentrations were calculated using prop-2-enyl-glucosinolate as standard for the external calibration curve and the response factor of each compound relative to prop-2-enyl-GL. Total GLs were determined from the sum of individual GLs. Each analysis was performed in duplicate. Desulfo-glucosinolates were identified by HPLC-ESI-MS2 using Agilent 1100 series (Agilent Technologies, Germany) operating in the positive ionization mode, based on the protonated molecular ions [M + H]^+^ and the fragment ions corresponding to [M + H-glucose]^+^, identified by Zimmermann et al. (2007).

The Supplementary Material S1 summarizes the GLs assessed in this study, indicating the belonging group, trivial name, IUPAC nomenclature, semi systematic names used in texts, and abbreviations used in tables and figures.

### Statistical Analysis

The Shapiro–Wilk and Kolmororov–Smirnov procedures were performed to verify that the data had a normal distribution, and the Levene, O’Brien and Bartlet tests were conducted to verify the homogeneity of variances. Then a multifactorial ANOVA of all data obtained was performed. Data were analyzed using PAR, Pi, and Phi as study factors. Means were compared using Tukey’s test (*P* ≤ 0.05%). In addition, regression analysis was performed to determine the relationship between flavonoids and nitrate, for which SAS 9.3 statistical software was used (SAS, 2011).

## Results

### Leaf Number and Biomass

**FIGURE 1:**
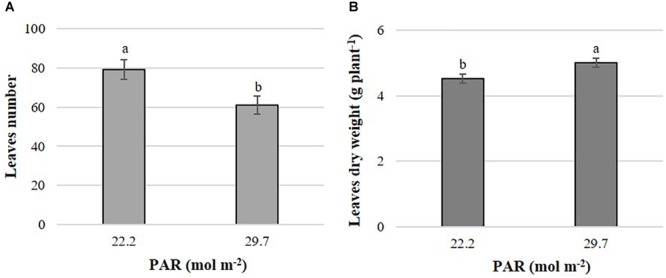
Effects of mean daily photosynthetically active radiation (PAR) on leaf number **(A)** and leaf dry matter **(B)** in *B. campestris* cv. Mibuna Early. Means ± SD with different letters in each subfigure indicate significant differences at *P* ≤ 0.05 (Tukey’s test).

The leaf number and dry biomass of leaves at harvest were affected by PAR only in *B. campestris* (Supplementary Materials S2, S3). Leaf number was higher in plants treated with lower PAR with an average value of approximately 79 leaves, exceeding in 18 leaves to the means observed in plants exposed to higher PAR (Figure 1A). Conversely, leaf dry matter was 10.6% higher in plants grown at higher PAR in comparison to those grown at lower PAR (Figure 1B).

### Flavonoid Concentrations

PAR significantly affected concentrations of quercetin, kaempferol, isorhamnetin, and total flavonoids in both *Brassica* species evaluated. In addition, Pi significantly influenced the concentrations of quercetin, isorhamnetin, and total flavonoids in *B. campestris*. In *B. juncea*, Pi only affected quercetin and cyanidin concentrations. Interestingly, PAR × Phi interaction modified all flavonoids quantified in *B. juncea* (Supplementary Material S4).

**FIGURE 2:**
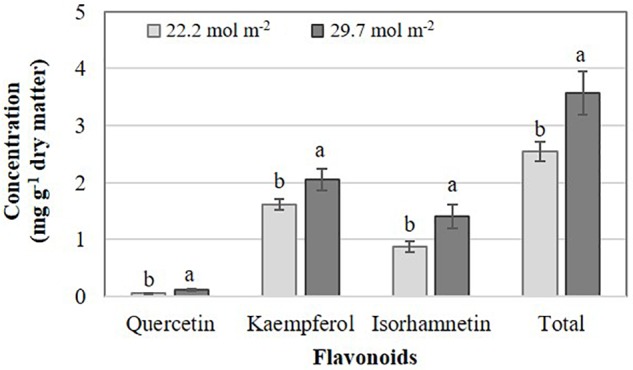
Effects of mean daily photosynthetically active radiation (PAR) on flavonoid concentration in *B. campestris* cv. Mibuna Early. Means ± SD with different letters in each variable analyzed indicate significant differences at *P* ≤ 0.05 (Tukey’s test).

The effects of PAR on flavonoid concentrations were different between *Brassica* species evaluated. In *B. campestris*, increasing PAR elevated the concentrations of quercetin, kaempferol, isorhamnetin, and total flavonoids by 121.4, 24.4, 61.1, and 40.3%, respectively, in comparison to the lower PAR applied (Figure 2). At high Phi, *B. juncea* exposed to higher PAR displayed higher quercetin concentrations in comparison to plants exposed to lower PAR, while no other interactions occurred. Plants exposed to lower PAR exhibited higher concentrations of kaempferol than those exposed to higher PAR independent of the Phi concentration applied. Isorhamnetin and total flavonoid concentrations were higher in plants exposed to lower PAR at any level of Phi tested. Cyanidin concentrations were the highest in plants exposed to higher PAR at high Phi (Table 1).

**Table 1:** Mean daily photosynthetically active radiation (PAR) and phosphite (Phi) affecting flavonoid concentration (mg g^-1^ dry matter) in *B. juncea* cv. Red Giant.

PAR (mol m^-2^)	Phi (mM)	Quercetin	Kaempferol	Isorhamnetin	Cyanidin	Total flavonoids
						
22.2	0.1	0.232 ± 0.02ab	2.50 ± 0.10a	1.36 ± 0.103a	0.66 ± 0.054ab	4.75 ± 0.24a
	0.5	0.227 ± 0.01ab	2.35 ± 0.12ab	1.23 ± 0.094ab	0.67 ± 0.044ab	4.48 ± 0.23ab
	2.5	0.194 ± 0.02b	2.27 ± 0.07ab	1.17 ± 0.053ab	0.57 ± 0.089b	4.20 ± 0.15abc
29.7	0.1	0.219 ± 0.01b	1.73 ± 0.18c	1.02 ± 0.057b	0.56 ± 0.059b	3.53 ± 0.26c
	0.5	0.250 ± 0.04ab	1.80 ± 0.14c	1.10 ± 0.092b	0.71 ± 0.137ab	3.87 ± 0.33bc
	2.5	0.287 ± 0.01a	2.04 ± 0.14bc	1.15 ± 0.069ab	0.85 ± 0.062a	4.33 ± 0.21ab

*Means ± SD with different letters in each column indicate statistically significant differences at *P* ≤ 0.05 (Tukey’s test).*

**FIGURE 3:**
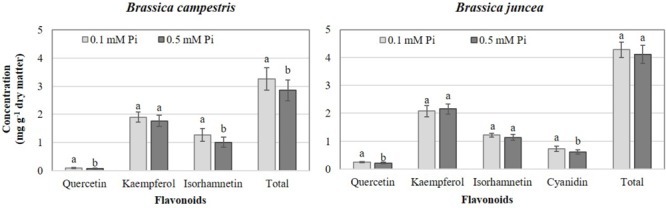
Effects of phosphate (Pi) on flavonoid concentration in two *Brassica* species. Means ± SD with different letters in each subfigure and different letters indicate significant differences at *P* ≤ 0.05 (Tukey’s test).

Deficient Pi in the nutrient solution resulted in a significant increase of total flavonoids at 14.3%, especially in *B. campestris*, which included increases of quercetin and isorhamnetin at 25.2 and 25.6% in comparison to sufficient Pi tested, respectively. However, in *B. juncea* deficient Pi only increased the concentrations of quercetin and cyanidin by 15.6 and 20.1%, respectively, in comparison to the application of sufficient Pi (Figure 3).

The PAR × Phi interaction was significant for flavonoids concentrations in *B. juncea* (Table 1). Quercetin, kaempferol, isorhamnetin, cyanidin and total flavonoids had their highest concentrations at low Phi and higher PAR. Conversely, at lower PAR, all flavonoids but cyanidin reduced their concentrations when Phi levels increased in the nutrient solution.

### Nitrate Concentrations

The *Brassica* species evaluated displayed different responses to the factors studied regarding nitrate concentrations in edible tissues. Between the two species, *B. campestris* showed stronger effects. Phi as main factor did not affect nitrate concentration in either species (Supplementary Material S5).

**FIGURE 4:**
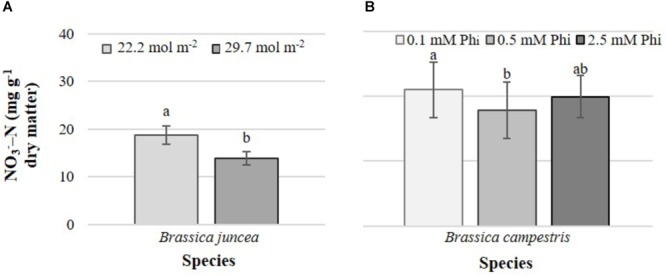
Effects of mean daily photosynthetically active radiation (PAR) and phosphite (Phi) on nitrate concentration in *B. juncea* cv. Red Giant **(A)** and *B. campestris* cv. Mibuna Early **(B)**, respectively. Means ± SD with different letters in each subfigure indicate significant differences at *P* ≤ 0.05 (Tukey’s test).

Increasing the PAR value from 22.2 to 29.7 mol m^-2^ decreased nitrate concentrations in edible tissues by 35.5%, but only in *B. juncea* (Figure 4A). Phi only affected nitrate concentrations in *B. campestris*, decreasing it by 15.3% in plants exposed to medium Phi, with respect to those exposed to low Phi (Figure 4B).

**FIGURE 5:**
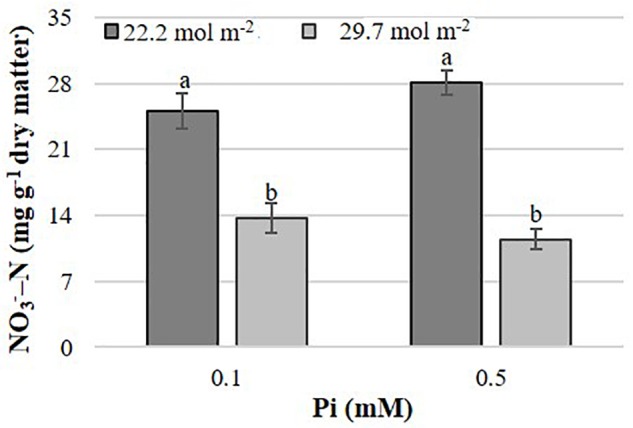
Effects of mean daily photosynthetically active radiation (PAR) and phosphate (Pi) on nitrate concentration in *B. campestris* cv. Mibuna Early. Means ± SD with different letters indicate significant differences at *P* ≤ 0.05 (Tukey’s test).

In *B. campestris*, the PAR × Pi interaction significantly affected the nitrate concentration in leaves (Figure 5). Nitrate concentration was generally higher in plants at lower PAR. At lower PAR it increased with increasing Pi but decreased at higher PAR.

The rest of the interactions had no significant effects on nitrate concentrations in ether species evaluated (Supplementary Material S5).

### Relationship Between Total Flavonoids and Nitrate Concentrations

**FIGURE 6:**
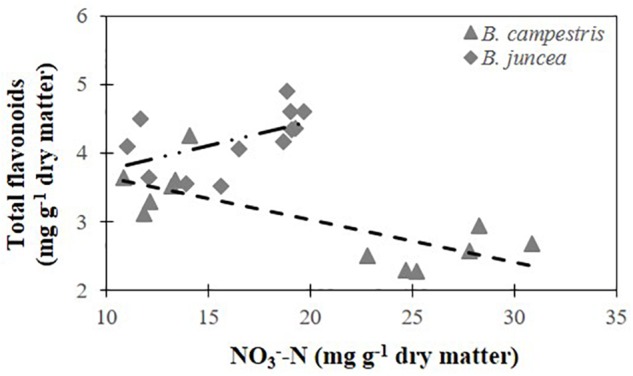
Relationship between total flavonoid concentration and nitrate concentration in two *Brassica* species. Total flavonoids in *B. juncea* cv. Red Giant = 3.040+ 0.071 ⋅ (NO_3_^-^-N) (*R*^2^= 0.279). Total flavonoids in *B. campestris* cv. Mibuna Early = 4.269 – 0.062 ⋅ (NO_3_^-^-N) (*R*^2^= 0.576^∗^). ^∗^Significant at *P ≤* 0.05.

A significant negative relationship between total flavonoids and nitrate concentrations was analyzed for *B. campestris* with *R*^2^= 0.576 (Figure 6). The same relationship was non-significant in *B. juncea* (*R*^2^= 0.279).

### Study Factors and Their Interactions Influencing Glucosinolate Concentrations

**FIGURE 7:**
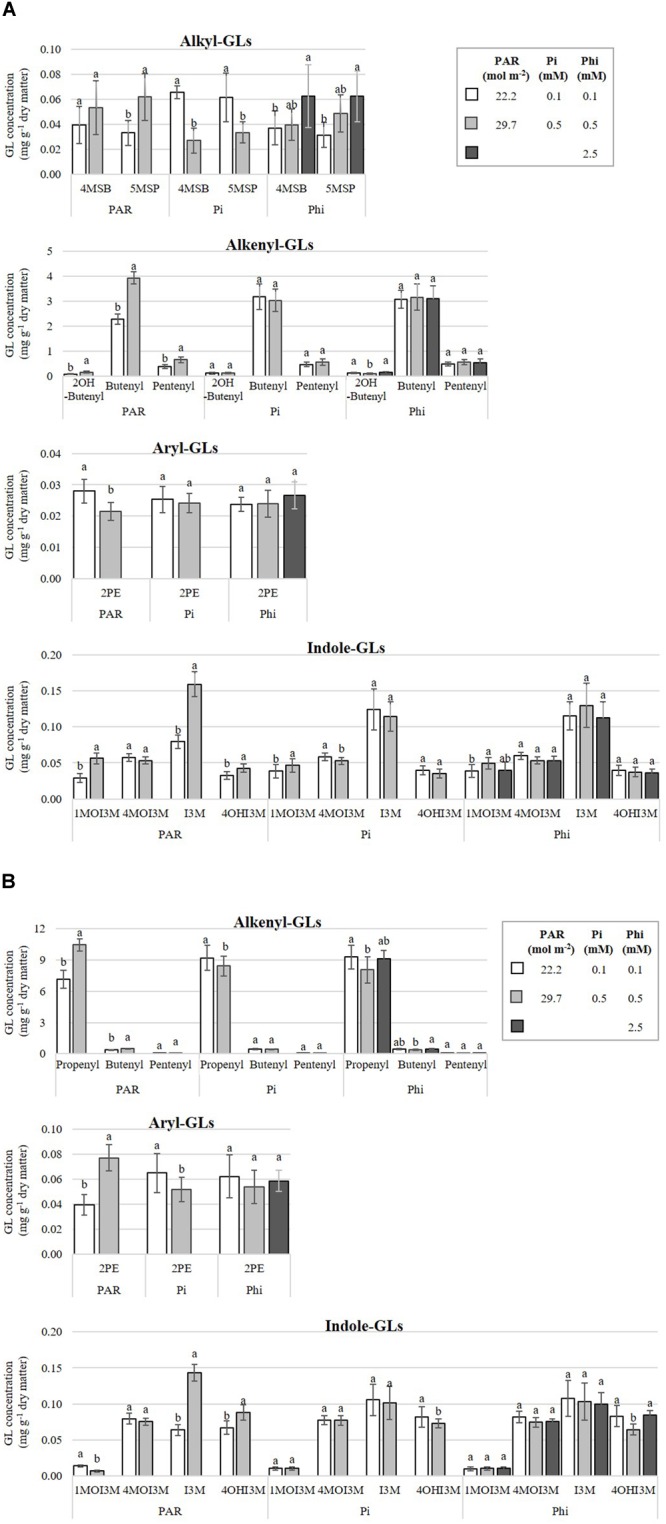
Influence of mean daily photosynthetically active radiation (PAR), phosphate (Pi), and phosphite (Phi) on glucosinolate (GL) concentrations. Bars ± SD with different letters in each subfigure indicate significant differences at *P* ≤ 0.05 (Tukey’s test). **(A)** *B. campestris* cv. Mibuna Early. **(B)** *B. juncea* cv. Red Giant. 4MSB, 4-methylsulfinylbutyl-GL; 5MSP, 5-methylsulfinylpentyl-GL; 2OH-Butenyl, 2-hydroxybut-3-enyl-GL; Butenyl, but-3-enyl-GL; Propenyl, prop-2-enyl-GL; Pentenyl, pent-4-enyl-GL; 2PE, 2-phenylethyl-GL; 1MOI3M, 1-methoxyindol-3-ylmethyl-GL; 4MOI3M, 4-methoxyindol-3-ylmethyl-GL; I3M, indol-3-ylmethyl-GL; 4OHI3M, 4-hydroxyindol-3-ylmethyl-GL.

Effects of PAR were significant in *B. campestris* for total alkyl-GL (only detected in this species), total alkenyl-GL, and the aryl-GL (2-phenylethyl) (Supplementary Material S6a). Among alkyl-GLs, plants exposed to higher PAR displayed concentrations of 5-methylsulfinylpentyl-GL 87.7% higher than plants exposed to lower PAR. Interestingly, plants exposed to higher compared with lower PAR exhibited 89.4% higher concentrations of the three alkenyl-GLs identified (2-hydroxybut-3-enyl-, but-3-enyl-, and pent-4-enyl-GL), and 96.1 and 101.1% higher concentrations of the indole-GLs, 1-methoxyindol-3-ylmethyl- and indol-3-ylmethyl-GL. Conversely, the higher PAR level significantly reduced the concentration of 2-phenylethyl-GL, which was 23.4% lower than that observed in plants grown under lower PAR level (Figure 7A).

Increasing the PAR level from 22.2 to 29.7 mol m^-2^ raised the concentration of indol-3-ylmethyl-GL and 4-hydroxyindol-3-ylmethyl-GL in *B. juncea.* However, at higher PAR, the concentration of 1-methoxyindol-3-ylmethyl-GL fell by 51.5% in comparison to lower PAR. Within the group of alkenyl-GLs, prop-2-enyl-GL (propenyl) and but-3-enyl-GL (butenyl) were increased by 52 and 30.4%, respectively (Supplementary Material S6b and Figure 7B).

In *B. campestris*, Pi-deficient plants increased the concentrations of two alkyl-GLs, namely 4-methylsulfinylbutyl-GL and 5-methylsulfinylpentyl-GL, by 144.3 and 64.3%, respectively, in comparison to Pi-sufficient plants. Among the indole-GLs, Pi differentially affected the concentrations of 1-methoxyindol-3-ylmethyl-GL, being reduced by 17.3% in Pi-deficient plants, in comparison to Pi-sufficient plants (Supplementary Material S6a and Figure 7A).

In *B. juncea*, Pi significantly increased the concentrations of prop-2-enyl-GL (9.2%), total alkenyl-GLs and the aryl-GL (2-phenylethyl-GL) comparing Pi-deficient with Pi-sufficient plants (Supplementary Material S6b). All other GLs evaluated in this species were also increased under Pi-deficiency (Figure 7B).

The concentrations of 1-methoxyindol-3-ylmethyl-GL, and 4-methoxyindol-3-ylmethyl-GL were significant influenced by Phi in *B. campestris* (Supplementary Material S6a). Regarding indole-GLs, plants exposed to medium Phi significantly increased by 28.3% the concentration of 1-methoxyindol-3-ylmethyl-GL, in comparison to plants treated with low Phi (Figure 7A).

In *B. juncea*, Phi only had effect on but-3-enyl-GL concentration (Supplementary Material S6b). Indeed, the highest concentrations were recorded in plants treated with high Phi. The concentrations were 18.9% higher in comparison to those in plants grown at medium Phi (0.5 mM) (Figure 7B).

The PAR × Pi interaction affected the concentrations of 2-hydroxybut-3-enyl-, 4-methoxyindol-3-ylmethyl-GL, and total indole-GLs in *B. campestris*. In this species, the PAR × Phi interaction predominantly affected the concentrations of 5-methylsulfinylpentyl-, 2-hydroxybut-3-enyl-, and but-3-enyl-GL. The Pi × Phi interaction only influenced the concentration of total alkyl-GLs, and 4-methylsulfinylbutyl-GL belonging to this group. The PAR × Pi × Phi interaction did not influence the concentrations of GLs in *B. campestris* (Supplementary Material S6a).

In *B. juncea*, the PAR × Pi interaction influenced only the concentration of 4-hydroxyindol-3-ylmethyl-GL. The concentrations of prop-2-enyl-, total alkenyl-, aryl- (2-phenylethyl-GL), indol-3-ylmethyl-, 4-hydroxyindol-3-ylmethyl-, and total-GLs, were affected by PAR × Phi. The Pi × Phi interaction significantly affected the indol-3-ylmethy-GL concentration. In the same way, the interaction PAR × Pi × Phi influenced indol-3-ylmethyl-GL, total indole-GLs and total-GLs (Supplementary Material S6b).

**FIGURE 8:**
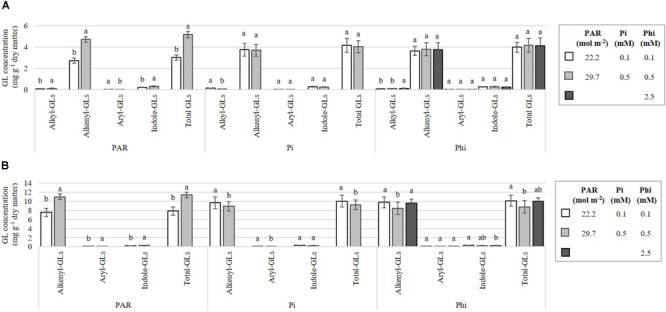
Influence of mean daily photosynthetically active radiation (PAR), phosphate (Pi), and phosphite (Phi) on the alkyl-, alkenyl-, aryl-, indole-, and total glucosinolate (GL) concentrations. Bars ± SD with different letters indicate significant differences at *P* ≤ 0.05 (Tukey’s test). **(A)** *B. campestris* cv. Mibuna Early. **(B)** *B. juncea* cv. Red Giant.

At higher compared with lower PAR, we observed a concomitant increase in the concentrations of the aryl-GL, alkyl-, alkenyl-, indole-, and total GLs in *B. campestris* (Figure 8A). In *B. juncea*, Pi-deficient plants had increased concentrations of alkenyl-GLs and the aryl-GL, by at least 8.8% compared to the values observed in Pi-sufficient plants (Figure 8B).

In *B. campestris* the concentrations of some GLs were significantly affected by the interactions PAR × Pi, PAR × Phi and Pi × Phi (Table 2). The interaction PAR × Pi × Phi was without a significant influence.

**Table 2:** Mean daily photosynthetically active radiation (PAR), phosphate (Pi), and phosphite (Phi) affecting glucosinolate (GL) concentrations in *B. campestris* cv. Mibuna Early.

PAR × Pi interaction				
PAR (mol m^-2^)	Pi (mM)	GL concentration (mg g^-1^ dry matter)		
		2OH-Butenyl	4MOI3M	Total indole-GLs
22.2	0.1	0.0777 ± 0.0081b	0.0345 ± 0.0032b	0.2059 ± 0.0084b
	0.5	0.0616 ± 0.0047b	0.0228 ± 0.0021b	0.1904 ± 0.0235b
29.7	0.1	0.1416 ± 0.0093a	0.0584 ± 0.0043a	0.2927 ± 0.0240a
	0.5	0.1715 ± 0.0158a	0.0540 ± 0.0034a	0.3312 ± 0.0202a
**PAR × Phi interaction**				
**PAR (mol m^-2^)**	**Phi (mM)**	**GL concentration (mg g^-1^ dry matter)**		
		**5MSP**	**2OH-Butenyl**	**Butenyl**

22.2	0.1	0.023 ± 0.004d	0.085 ± 0.018cd	2.465 ± 0.201b
	0.5	0.039 ± 0.015bc	0.059 ± 0.010d	2.189 ± 0.187b
	2.5	0.036 ± 0.008bc	0.064 ± 0.010d	2.155 ± 0.241b
29.7	0.1	0.039 ± 0.013bc	0.148 ± 0.017b	3.651 ± 0.198a
	0.5	0.058 ± 0.014ab	0.117 ± 0.011bc	4.116 ± 0.208a
	2.5	0.088 ± 0.021a	0.205 ± 0.027a	4.010 ± 0.246a
**Pi × Phi interaction**				
**Pi (mM)**	**Phi (mM)**	**GL concentration (mg g^-1^ dry matter)**	
		**4MSB**	**Total alkyl-GLs**

0.1	0.1	0.0556 ± 0.0137b	0.0924 ± 0.0251bc
	0.5	0.0429 ± 0.0095b	0.1041 ± 0.0257b
	2.5	0.0984 ± 0.0240a	0.1841 ± 0.0381a
0.5	0.1	0.0183 ± 0.0040b	0.0438 ± 0.0059c
	0.5	0.0359 ± 0.0156b	0.0715 ± 0.0201bc
	2.5	0.0264 ± 0.0052b	0.0649 ± 0.0121bc

*Means ± SD with different letters in each column and analyzed variable indicate significant differences at *P* ≤ 0.05 (Tukey’s test). 2OH-Butenyl, 2-hydroxybut-3-enyl-GL; 4MOI3M, 4-methoxyindol-3-ylmethyl-GL; 5MSP, 5-methylsulfinylpentyl-GL; Butenyl, but-3-enyl-GL; 4MSB, 4-methylsulfinylbutyl-GL.*

Regarding PAR × Pi interaction, both levels of Pi at higher PAR significantly increased the concentrations of 2-hydroxybut-3-enyl-, 4-methoxyindol-3-ylmethyl-GL and total indole-GLs (Table 2).

All three Phi levels combined with higher PAR significantly increased the concentrations of but-3-enyl-GL. The concentration of 5-methylsulfinylpentyl-GL reduced as the Phi did, independent of the PAR level. Nevertheless, such decrease was more evident in plants exposed to higher PAR (55.7%) in comparison to that observed in plants exposed to lower PAR (36.1%). Concentration of 2-hydroxybut-3-enyl-GL with increasing Phi in the nutrient solution at lower PAR, whereas it increased at higher PAR (Table 2).

The Pi × Phi interaction significantly affected the concentrations of 4-methylsulfinylbutyl-GL and total alkyl-GLs (Table 2). In both cases, the highest levels were observed in Pi-deficient plants in combination with the high Phi concentration.

In *Brassica juncea*, all interactions between factors were significant at least for one GL analyzed (Tables 3, 4).

**Table 3:** Mean daily photosynthetically active radiation (PAR) and phosphite (Phi) affecting glucosinolate (GL) concentrations in *B. juncea* cv. Red Giant.

PAR × Phi interaction				
		GL concentration (mg g^-1^ dry matter)		
PAR (mol m^-2^)	Phi (mM)	Propenyl	Alkenyl-GLs	2PE
22.2	0.1	7.609 ± 0.819bc	8.018 ± 0.870bc	0.0363 ± 0.005de
	0.5	5.824 ± 0.550c	6.148 ± 0.583c	0.0291 ± 0.002e
	2.5	8.105 ± 0.770b	8.558 ± 0.803b	0.0527 ± 0.011cd
29.7	0.1	10.998 ± 0.673a	11.502 ± 0.690a	0.0880 ± 0.015a
	0.5	10.300 ± 0.586a	10.797 ± 0.607a	0.0787 ± 0.004ab
	2.5	10.128 ± 0.425a	10.649 ± 0.444a	0.0646 ± 0.005bc

*Means ± SD with different letters in the column indicate significant differences at *P* ≤ 0.05 (Tukey’s test). Propenyl, prop-2-enyl-GL; 2PE, 2-phenylethyl-GL.*

**Table 4:** Mean daily photosynthetically active radiation (PAR), phosphate (Pi), and phosphite (Phi) affecting glucosinolate (GL) concentrations in *B. juncea* cv. Red Giant.

PAR (mol m^-2^)	Pi (mM)	Phi (mM)	GL concentration (mg g^-1^ dry matter)			
			I3M	4OHI3M	Total indole-GLs	Total-GLs
22.2	0.1	0.1	0.060 ± 0.007d	0.057 ± 0.010cd	0.218 ± 0.026cd	8.221 ± 1.080bcd
		0.5	0.067 ± 0.009d	0.048 ± 0.003d	0.202 ± 0.020d	6.390 ± 0.886d
		2.5	0.081 ± 0.006cd	0.093 ± 0.004ab	0.262 ± 0.009bcd	10.018 ± 0.789abc
	0.5	0.1	0.063 ± 0.006d	0.079 ± 0.007bcd	0.247 ± 0.023bcd	8.352 ± 0.837bcd
		0.5	0.047 ± 0.006d	0.055 ± 0.002cd	0.193 ± 0.013d	6.358 ± 0.213 d
		2.5	0.063 ± 0.001d	0.070 ± 0.003bcd	0.221 ± 0.005cd	7.686 ± 0.230cd
29.7	0.1	0.1	0.153 ± 0.015ab	0.119 ± 0.016a	0.356 ± 0.023a	12.819 ± 0.756a
		0.5	0.161 ± 0.012a	0.080 ± 0.007bcd	0.326 ± 0.022ab	11.505 ± 0.762ab
		2.5	0.112 ± 0.003bc	0.093 ± 0.005ab	0.288 ± 0.015abc	11.120 ± 0.603ab
	0.5	0.1	0.155 ± 0.005a	0.078 ± 0.007bcd	0.309 ± 0.002ab	11.032 ± 0.323ab
		0.5	0.137 ± 0.010ab	0.074 ± 0.003bcd	0.288 ± 0.019abc	10.860 ± 0.525abc
		2.5	0.143 ± 0.001ab	0.083 ± 0.02bc	0.313 ± 0.005ab	10.907 ± 0.333abc

*Means ± standard deviation with different letters in the column indicate significant differences at *P* ≤ 0.05 (Tukey’s test). I3M, indol-3-ylmethyl-GL; 4OHI3M, 4-hydroxyindol-3-ylmethyl-GL.*

Regarding the PAR × Phi interaction, the lower level of PAR combined with the medium Phi significantly reduced the concentrations of prop-2-enyl-GL and alkenyl-GLs, in comparison to the concentrations of those GLs found in plants exposed to high Phi. In plants exposed to higher PAR and high Phi the concentration of 2-phenylethyl-GL was significantly reduced in comparison to plants treated with low Phi. Furthermore, plants exposed to lower PAR displayed lower concentrations of 2-phenylethyl-GL, independent of the Phi concentrations tested (Table 3).

Significant effects of the PAR × Pi × Phi interaction on GL concentrations are presented in Table 4. The highest concentrations of indol-3-ylmethyl-GL, total indole-, and total-GLs were recorded in plants receiving higher PAR, independent of the Pi and Phi supply levels. However, at lower PAR, higher Phi and deficient Pi, similar concentrations of 4-hydroxyindol-3-ylmethyl- and total-GLs were reached.

## Discussion

Light affects photosynthesis, phototropism and morphogenetic processes, and consequently, it has a pivotal role in plant metabolism, growth and development. Importantly, light quality and intensity, direction and duration impact plant primary and secondary metabolism (Debiasi et al., 2003). Indeed, the intensity of photosynthetically active radiation (PAR) during the day determines the rate of mobilization of organic compounds, while its amount impacts the extent of CO_2_ absorption at night (Diez et al., 2017). Carbon fixation and biosynthesis of primary and secondary metabolites are crucial for cell expansion and division, which are controlled at the molecular level. Although in *B. juncea* it has been reported that the expression of the gene *BjAPY2* (involved in the expansion of edible stems) was higher under short-day photoperiod (8 h/16 h) than under long-day photoperiod (16 h/8 h) (Cao et al., 2015), this species was neither affected by the study factors, nor by their interactions on growth characteristics evaluated under our experimental conditions. In *B. campestris*, only PAR influenced leaf number and dry matter (Figure 1).

In kale (*B. oleracea* var. *sabellica*), Neugart et al. (2016) reported a higher number of genes regulated by light than by temperature. Those genes were mainly related to protein degradation (in response to light), phytohormone metabolism (in response to temperature), and secondary metabolism (in response to both treatments). The expression patterns of genes involved in the biosynthesis of flavonoids were correlated to the structure-dependent response of those metabolites to alterations in either light or temperature. Under our experimental conditions, differential effects of PAR on the synthesis of flavonoids were observed between the two *Brassica* species evaluated. In general, the biosynthesis of flavonoids (quercetin, kaempferol, isorharmnetin and total) in *B. campestris* was associated with a higher PAR level evaluated (Figure 2). Coincidentally, in kale (*B. oleracea* var. *sabellica*), the highest light intensity evaluated (14.4 mol m^-2^ d^-1^) increased the foliar concentration of flavonols, including quercetin and kaempferol, in comparison to the means showed by plants exposed to lower light intensity (3.6 mol m^-2^ d^-1^) (Neugart et al., 2016). Conversely, in spiked pepper (*Piper aduncum*), the lowest concentration of flavonoids was recorded in plants grown under 100% natural irradiance (value of 15.4 W m^-2^ seems to be erroneously given), in comparison to those grown at 50 and 70% natural irradiance. These differential responses observed among plant genotypes suggest the existence of different mechanisms developed by plants to protect themselves against irradiation (Ventorim et al., 2014). Indeed, in Chinese cabbage (*B. campestris* ssp. *Chinensis Makino*), the antioxidant activity of guaiacol peroxidase, catalase, and superoxide dismutase transiently increased in treatments with 75, 50, and 25% of the normal light intensity, especially 5 days after treatment (Zhu et al., 2017). Thus, although it has been documented that external factors, including light intensity, have an influence on the composition of secondary metabolites in plants, the main determinant of the metabolic profiles in plant tissues is the genotype (Zoratti et al., 2014). Among different rice genotypes remarkable variations in PAR transmission percentage with respect to variation in canopy shape were first observed after an early heading stage and continued thereafter (Yusoff and Zainol, 1989). Moreover, interactions among genotypes and N-management practices produced different PAR interception between two maize genotypes studied (Ghosh et al., 2017). Additionally, PAR absorption also differed among coffee genotypes (Mejía-Montoya et al., 2013). Indeed, PAR induced phytochemical changes in two *Brassica* species, which were different according to the genotypes tested (Fallovo et al., 2009, 2011). Coincidentally, Ghasemzadeh et al. (2010) as well as Pazuki et al. (2017) reported similar results. Taken together, our findings are in full agreement with those previously reported.

Various environmental conditions including N and P deficiencies bring about increases in flavonoid concentrations (Stewart et al., 2001). This response is in full agreement with our findings since the concentration of quercetin and isorhamnetin in *B. campestris* and of quercetin and cyanidin in *B. juncea* increased in Pi-deficient plants (Figure 3). It is well known that Phi suppresses the coordinated expression of genes under phosphate starvation, leading to negative effects on plant growth and metabolism (McDonald et al., 2001; Varadarajan et al., 2002). Under our experimental conditions, however, Phi did not impair the concentration of flavonoids in tissues of either species evaluated.

Flavonoid biosynthesis was stimulated at increasing Phi concentration in the nutrient solution only at higher PAR (Table 1). In potato (*Solanum tuberosum*) the expression levels of *F3H*, a gene involved in flavonoid synthesis, increased in UV-B-stressed plants only when pre-treated with potassium Phi, while Phi may prevent oxidative damages caused by UV-B light by increasing the enzymatic activity (Oyarburo et al., 2015). Phi has been classified as an emergent biostimulant in horticulture. As such, Phi may display hormetic effects in plants, which means that at low doses it induces a beneficial effect and at high doses it produces a toxic effect (Trejo-Téllez and Gómez-Merino, 2018). At the physiological level, hormesis can be translated as an adaptive response of an organism to a low level of such factor, accompanied by overcompensation, when the homeostasis readjustment has been interrupted (Calabrese and Blain, 2009; Vargas-Hernández et al., 2017). This allows the organism to acclimate to its new environment. Indeed, the level of eustress (beneficial stress) or distress (harmful stress) toward the same factor (e.g., a biostimulant such as Phi) is not always the same due to the process of adaptation of the plants, which must be taken into account when establishing a strict difference between low dose and high dose of a hormetic factor. In order Phi to induce eustress, plants must be established in the presence of sufficient Pi. Interestingly, under our experimental conditions, fluctuations between Pi and Phi did not influence growth parameters in any *Brassica* species evaluated (Supplementary Materials S2, S3). Hence, one can assume that the levels of Pi and Phi caused eustress stimulating secondary metabolite synthesis as observed. Apart from its effect on plant metabolism, Phi has been proved to enhance important traits including plant growth and development, nutrition efficiency, abiotic stress tolerance, yield and crop quality in the presence of sufficient Pi (Estrada-Ortiz et al., 2011, 2012; Rossall et al., 2016).

Previously, Fallovo et al. (2009, 2011) reported that N supply and PAR differentially affect phytochemical composition of *Brassica* species. For instance, when N was supplied as 100% NH_4_^+^ under medium PAR (i.e., 6.8 mol m^-2^ day^-1^), the highest concentration of GLs as well as high levels of carotenoids in the leaves of both *Brassica* species were observed. However, the 100% NH_4_^+^ supply under low (5.0 mol ⋅ m^-2^ ⋅ day^-1^) and medium (6.8 mol ⋅ m^-2^ ⋅ day^-1^) PAR levels resulted in low concentrations of flavonoids. Our results are in accordance to previously reported studies, and importantly, they are supported by strict statistical analyses and mean comparisons.

The light activation of nitrate reductase occurs at both the transcriptional and posttranslational levels (Lillo and Appenroth, 2001). Since nitrate reductase catalyzes the reduction of nitrate into nitrite, a higher activity of this enzyme is expected to lower the levels of nitrate in plant tissues. While light renders nitrate reductase active, darkness results in inactivation of this enzyme (Lillo et al., 2004). Under our experimental conditions, PAR significantly affected the foliar nitrate concentrations in *B. juncea*; when PAR level increased, the nitrate concentrations in plant tissues decreased (Figure 4A), which indicates that a higher PAR induced a stronger activity of nitrate reductase. According to Fallovo et al. (2009), in *B. rapa* subsp. *nipposinica* var. *chinoleifera* and *B. juncea* exposed to three different PAR treatments (5.0 mol m^-2^ d^-1^, 6.8 mol m^-2^ d^-1^ and 9.0 mol m^-2^ d^-1^), low and high PAR levels increased the nitrate concentration in leaves of both crops compared to medium PAR level.

Nitrogen can increase P uptake in plants leading to a positive interaction between N and P nutrition (Fageria, 2001). In tomato (*S. lycopersicum*), N concentration in plant tissues decreased with increasing P limitation induced by the addition of Phi (de Groot et al., 2003). It is well documented that Phi is able to disrupt Pi-starvation responses in plants (Ticconi et al., 2001; Varadarajan et al., 2002), which may explain the results observed in tomato. Indeed, Phi prevents the activation of many genes involved in Pi-starvation responses thus altering P nutrition. According to Danova-Alt et al. (2008), Phi inhibits Pi uptake in a competitive manner and induces a range of physiological and developmental responses by altering the homeostasis of Pi (Kobayashi et al., 2006; Berkowitz et al., 2013). In turn, Phi uptake is strongly and competitively inhibited in the presence of Pi (Pratt et al., 2009; Jost et al., 2015). Under our experimental conditions, *B. campestris* plants exposed to medium Phi level only exhibited negative effects on nitrate concentration, in comparison to plants exposed to low Phi level. Nonetheless, there were no significant differences between plants exposed to low and high Phi concentrations (Figure 4B). Conversely, in oat (*Avena sativa*), Phi did not impair N status, though it did reduce plant growth as well as magnesium and sulfur nutrition in a more pronounced manner than Pi-deficiency, which suggests toxic effects of Phi itself (Zambrosi, 2016).

In *B. campestris*, the PAR × Pi interaction showed that PAR significantly affected the concentrations of nitrate in plant tissues, independent of the Pi level supplied in the nutrient solution (Figure 5), which confirms the direct effect of PAR on the induction of the activity of the nitrate reductase enzyme (Gómez et al., 1998).

In apple (*Malus domestica*) trees, the supply of high N concentrations reduced flavonoid production in leaves, which was attributed to a decrease the enzymatic activity of phenylalanine ammonia lyase (PAL) when N availability increases, thus causing the decrease in flavonoid concentration. PAL is the first enzyme of the phenylpropanoid pathway, which provides one of the precursors for flavonoid formation and is responsible for the conversion of phenylalanine to cinnamic acid (Strissel et al., 2005). Likewise, in *B. campestris*, high nitrate concentrations in leaves were associated with a decrease in the flavonoid concentration, though the coefficient of determination was too low to certainly attribute the decrease in flavonoids to the nitrate present in leaves. In *B. juncea*, flavonoids were not affected by the increase in leaf nitrate concentration because the regression analyses did not render significant results for the relationship (Figure 6).

We observed a greater diversity of GLs in *B. campestris* in comparison to *B. juncea* (Figure 7). In total, two alkyl-GLs (4-methylsulfinylbutyl- and 5-methylsulfinylpentyl-GL), three alkenyl-GLs (but-3-enyl-, pent-4-enyl-, and 2-hydroxybut-3-enyl-GL), an aryl-GL (2-phenylethyl-GL) and four indole-GLs (indol-3-ylmethyl-, 4-hydroxyindol-3-ylmethyl-, 4-methoxyindol-3-ylmethyl-, and 1-methoxyindol-3-ylmethyl-GL) were identified in the former species. Nonetheless, *B. juncea* displayed 50% higher concentrations of GLs than *B. campestris* did.

Alkenyl-GLs (also known as aliphatic GLs) belong to the most abundant group of GLs found in *B. juncea* (Figure 8B). Among this group, prop-2-enyl-GL was found in the greatest concentration (Figure 7B). Coincidentally, Fallovo et al. (2011) and Tong et al. (2014) also reported that 90% of total GLs identified in plant tissues corresponded to alkenyl-GLs. Under our experimental conditions, the high PAR level rendered nearly double the concentrations of GLs reported by Fallovo et al. (2011) in *B. juncea*, with PAR levels of 5.0, 6.8, and 9.0 mol m^-2^ (Figure 7B, 8B).

In *B. campestris*, the most abundant GL was but-3-enyl-GL, which belongs to the group of alkenyl-GLs, followed by the indole-GLs, alkyl-GLs and finally the aryl-GL, 2-phenylethyl-GL (Figure 7A, 8B). In several subspecies belonging to *B. campestris*, but-3-enyl-GL (gluconapin) has been reported as the most abundant aliphatic GL (Chen et al., 2008), which is in full agreement with our results. Similarly, in *B. campestris* subsp. *pekinensis*, Verkerk et al. (2009) reported that pent-4-enyl-GL (glucobrassicanapin) is the most abundant of the aliphatic GLs.

In both species, PAR significantly affected GL concentrations (Table 2). Both in *B. campestris* and *B. juncea*, the higher PAR level evaluated increased the concentrations of individual (Figure 7) and total GLs (Figure 8). Low PAR levels reduce the GL concentrations due to a decrease in the enzyme flavin-containing monooxygenase, which catalyzes the formation of aliphatic aldoxime, a key compound in the formation of aliphatic GLs (Wallsgrove and Bennet, 1995). In canola (*B. napus*), it has also been reported that reduced PAR results in a decrease in the GL concentration (Wallsgrove and Bennet, 1995). Moreover, in *Arabidopsis thaliana*, the levels of GLs and glutathione were found to be higher during the day than during the night, which coincides with the variation of sulfur uptake as well as the activity of the key enzyme of the sulfur assimilation pathway, adenosine 5′-phosphosulfate reductase (APR) (Huseby et al., 2013). Similarly, broccoli (*B. oleracea*) sprouts grown in the light synthesized 33% more GLs in comparison to sprouts grown in the darkness (Pérez-Balibrea et al., 2008), which further demonstrates that light stimulates GLs biosynthesis in *Brassica* species. Coincidentally, during seedling development of Chinese cabbage (*B. rapa* subsp. *pekinensis*), transcription levels of almost all transcription factors involved in the biosynthesis of GLs (i.e., *Dof1.1, IQD1-1, MYB28, MYB29, MYB34, MYB51*, and *MYB122*, and their isoforms) under light conditions were higher than under dark conditions, while total GLs contents under light conditions were also higher, which further demonstrates that light affects the levels of GLs (Kim et al., 2014). Conversely, in cabbage (*B. oleracea* var. *capitata*), total and individual GLs in the roots and in the aerial part showed the highest concentrations in the dark cycle, at 02:00 h and 22:00 h, respectively, while the lowest levels were during the light cycle, mainly at 18:00 h. Regardless of the link that seems to exist between light and the biosynthesis of GLs, their total content of GLs often fluctuates more than the gene expression, and elevated levels of GLs can be detected during the dark period when the genes have low expression levels (Rosa, 1997; Klein et al., 2006; Schuster et al., 2006; Huseby et al., 2013).

Phosphate concentration in the nutrient solution significantly affected alkyl-GL concentrations in *B. campestris*. Indeed, in Pi-deficient plants those GLs displayed higher concentrations as compared with Pi-sufficient plants (Figure 7A, 8A). This trend was also observed in *B. juncea* when significant effects of Pi on GL biosynthesis were detected (Figure 7B, 8B). Coincidentally, in rocket salad (*Eruca sativa*), Chun et al. (2017) found that applications of N and P at low concentrations, or higher concentrations of potassium enhanced the synthesis of total GLs; in particular 5 and 2 mM N and P possessed much higher levels of several types of aliphatic GLs than other nutrient concentrations tested. On the contrary, in yellow mustard (*Sinapis alba*) and oilseed radish (*Raphanus sativa*), P effects on GLs producing ionic or isothiocyanates were relatively insignificant (Brown et al., 2008).

Under our experimental conditions, high levels of Phi increased alkyl-GLs in *B. campestris* (Figure 7A, 8A), while in *B. juncea* the highest concentration of Phi tested enhanced the concentration of but-3-enyl-GL (alkenyl-GL) (Figure 7B). This confirms that Phi and similar biostimulants can be used to enhance bioactive compounds as GLs (Gómez-Merino and Trejo-Téllez, 2015, 2016), contributing to provide horticultural crops rich in bioactive compounds imparting health benefits for the consumer. Nowadays, nutraceutics are becoming more significant for human health (Singh et al., 2017).

Pi deficiency at a light intensity of 640 μmol m^-2^ s^-1^, increase GL concentrations, particularly of aliphatic- and indole-GLs, while the concentrations of free amino acids was increased by supplying the plant with low Pi concentrations (Yang et al., 2009). Among the increased amino acids is methionine, which is a precursor of aliphatic-GLs; tyrosine and phenylalanine also increased and function as precursors of aromatic-GLs. Interestingly, Ciereszko et al. (2005) reported an additive effect between Pi deficiency and light/dark conditions in gene expression, enhancing UDPG pyrophosphorylase activity and sugars concentrations, especially in Pi-deficient plants. During the GL biosynthesis, the thiohydroximic acid is released from the *S*-alkylthiohydroximate by action of a cysteine (C-S) lyase; the thiohydroximic acid is glucosylated by the action of UDPG, thus forming desulfo-glucosinolates (Wittstock and Halkier, 2002). Consequently, the accumulation of GLs stimulated by Phi observed in *B. juncea* may be a response to Pi deficiency (Table 3).

Summarizing, as a biostimulant, Phi has been proved to enhance not only production and productivity of diverse crops, but also the quality of products. In peaches, Phi enhanced both sugar content and soluble solid content, while in raspberry, Phi improved fruit firmness (an invaluable commercial trait leading to premium pricing of the product) (Achary et al., 2017). Nonetheless, precise studies on the economic trade-offs are lacking. Importantly, no negative effects on taste and odor of plants and plant products have been found in response to the application of Phi at rates not higher than 4.0 L ha^-1^ (content of active ingredient: nominal 504 g L^-1^ phosphonic acid equivalents) (Evaluator, 2017). However, sensory profiles of Phi-treated Brassicas remain a daunting task.

## Conclusion

It was observed in this study that Pi deficiency has a positive effect on the accumulation of some flavonoids and GLs, mainly under higher PAR; it was also observed that a lower PAR level tend to decrease flavonoid and GL concentrations. Since Phi is not metabolized by the plant, applying it in the nutrient solution tends to increase Pi deficiency; therefore, it favors the increase of some flavonoids and GLs as a possible defense mechanism for coping with stress. However, a balanced application of Pi and Phi to enhance flavonoid and GLs may be difficult because it conflicts with an adequate yield in horticultural production, if not properly scheduled to fulfill the Pi requirements of crop plants in order to positively stimulate physiological processes. Since a number of transcription factors and mRNAs have proved to be involved in the biosynthesis of GLs, their activity should be measured in future studies in order to analyze their activity in response to PAR, Pi, Phi and their interactions. Moreover, how such genes interact with other molecules (i.e., other genes, transcripts, proteins, or metabolites) to regulate the biosynthesis and degradation of flavonoids and nitrate in response to PAR, Pi, and Phi changes remains to be elucidated. New innovative techniques and their use in omics research (e.g., genomics, transcriptomics, metabolomics, proteomics, and interactomics) will be of paramount importance in achieving this goal and improving nutraceutic quality in *Brassica* species.

## Author Contributions

DS and LT-T designed the study. EE-O performed the experiments in greenhouse and the measurements in laboratory. LT-T carried out the statistical analyses and wrote the first draft of the manuscript. FG-M provided inputs for the study and edited the manuscript. AK and CB performed part of the analyses in laboratory and revised and edited the manuscript. All authors have given final approval for this version of the manuscript.

## Conflict of Interest Statement

The authors declare that the research was conducted in the absence of any commercial or financial relationships that could be construed as a potential conflict of interest.
