# Supplementary material for: Flavonoid, Nitrate and Glucosinolate Concentrations in Brassica Species Are Differentially Affected by Photosynthetically Active Radiation, Phosphate and Phosphite
Source: Front Plant Sci. 2019 Mar 27;10:371. doi: 10.3389/fpls.2019.00371 (PMC6445887; doi:10.3389/fpls.2019.00371)
Supplement: Supplementary file 1 [file Table_1.DOCX]

**Supplementary Material S1.** Glucosinolates (GLs) assessed in this study.

| **Glucosinolate group** | **Trivial name** | **IUPAC nomenclature** | **Semi systematic name** | **Abbreviation** |
| --- | --- | --- | --- | --- |
| Alkyl-GLs | glucoraphanin | [(2S,3R,4S,5S,6R)-3,4,5-trihydroxy-6-(hydroxymethyl)oxan-2-yl] (1E)-5-methylsulfinyl-N-sulfooxypentanimidothioate | 4-methylsulfinylbutyl-GL | 4MSB |
|  | glucoalyssin | [(2S,3R,4S,5S,6R)-3,4,5-trihydroxy-6-(hydroxymethyl)oxan-2-yl] (1Z)-6-methylsulfinyl-N-sulfooxyhexanimidothioate | 5-methylsulfinylpentyl-GL | 5MSP |
| Alkenyl-GLs | progoitrin | [(2S,3R,4S,5S,6R)-3,4,5-trihydroxy-6-(hydroxymethyl)oxan-2-yl] (3R)-3-hydroxy-N-sulfooxypent-4-enimidothioate | 2-hydroxybut-3-enyl-GL | 2OH-Butenyl |
|  | gluconapin | [(2S,3R,4S,5S,6R)-3,4,5-trihydroxy-6-(hydroxymethyl)oxan-2-yl] N-sulfooxypent-4-enimidothioate | But-3-enyl-GL | Butenyl |
|  | sinigrin | [(2S,3R,4S,5S,6R)-3,4,5-trihydroxy-6-(hydroxymethyl)oxan-2-yl] (1Z)-N-sulfooxybut-3-enimidothioate | Prop-2-enyl-GL | Propenyl |
|  | glucobrassicanapin | [(2S,3R,4S,5S,6R)-3,4,5-trihydroxy-6-(hydroxymethyl)oxan-2-yl] N-sulfooxyhex-5-enimidothioate | Pent-4-enyl-GL | Pentenyl |
| Aryl-GL | gluconasturtiin | [(2S,3R,4S,5S,6R)-3,4,5-trihydroxy-6-(hydroxymethyl)oxan-2-yl] 3-phenyl-N-sulfooxypropanimidothioate | 2-phenylethyl-GL | 2PE |
| Indole-GLs | neoglucobrassicin | [(2S,3R,4S,5S,6R)-3,4,5-trihydroxy-6-(hydroxymethyl)oxan-2-yl] (1Z)-2-(1-methoxyindol-3-yl)-N-sulfooxyethanimidothioate | 1-methoxyindol-3-ylmethyl-GL | 1MOI3M |
|  | 4-methoxy-glucobrassicin | [(2S,3R,4S,5S,6R)-3,4,5-trihydroxy-6-(hydroxymethyl)oxan-2-yl] (1Z)-2-(4-methoxy-1H-indol-3-yl)-N-sulfooxyethanimidothioate | 4-methoxyindol-3-ylmethyl-GL | 4MOI3M |
|  | glucobrassicin | [(2S,3R,4S,5S,6R)-3,4,5-trihydroxy-6-(hydroxymethyl)oxan-2-yl] 2-(1H-indol-3-yl)-N-sulfooxyethanimidothioate | Indol-3-ylmethyl-GL | I3M |
|  | 4-hydroxy-glucobrassicin | [(2S,3R,4S,5S,6R)-3,4,5-trihydroxy-6-(hydroxymethyl)oxan-2-yl] (1Z)-2-(4-hydroxy-1H-indol-3-yl)-N-sulfooxyethanimidothioate | 4-hydroxyindol-3-ylmethyl-GL | 4OHI3M |

Abbreviations are used in Tables and Figures. Semi systematic names are used in the text.
