# Supplementary material for: Flavonoid, Nitrate and Glucosinolate Concentrations in Brassica Species Are Differentially Affected by Photosynthetically Active Radiation, Phosphate and Phosphite
Source: Front Plant Sci. 2019 Mar 27;10:371. doi: 10.3389/fpls.2019.00371 (PMC6445887; doi:10.3389/fpls.2019.00371)
Supplement: Supplementary file 3 [file Table_3.DOCX]

**Supplementary Material S3.** Statistical significance (*P*) of mean daily photosynthetically active radiation (PAR), phosphate (Pi), phosphite (Phi), and their interactions on dry matter in two *Brassica* species. Tukey’s test, ns= not significant and * significant at *P* ≤ 0.05.

| **Study factors and interactions** | ***Brassica campestris*** | ***Brassica juncea*** |
| --- | --- | --- |
| PAR | <0.0001 * | 0.9566 ns |
| Pi | 0.6175 ns | 0.4813 ns |
| Phi | 0.5122 ns | 0.5941 ns |
| PAR × Pi | 0.2180 ns | 0.3318 ns |
| PAR × Phi | 0.6151 ns | 0.4417 ns |
| Pi × Phi | 0.0504 ns | 0.5020 ns |
| PAR × Pi × Phi | 0.6151 ns | 0.5463 ns |
