# Supplementary material for: Flavonoid, Nitrate and Glucosinolate Concentrations in Brassica Species Are Differentially Affected by Photosynthetically Active Radiation, Phosphate and Phosphite
Source: Front Plant Sci. 2019 Mar 27;10:371. doi: 10.3389/fpls.2019.00371 (PMC6445887; doi:10.3389/fpls.2019.00371)
Supplement: Supplementary file 4 [file Table_4.DOCX]

**Supplementary Material S4.** Statistical significance (*P*) of mean daily photosynthetically active radiation (PAR), phosphate (Pi), phosphite (Phi), and their interactions on flavonoid concentration in two *Brassica* species. Tukey’s test, ns= not significant and * significant at *P* ≤ 0.05.

|  | ***Brassica campestris*** | | | | | | | |
| --- | --- | --- | --- | --- | --- | --- | --- | --- |
| **Study factors and their interactions** | **Quercetin** | | **Kaempferol** | | **Isorhamnetin** | | **Total flavonoids** | |
| PAR | <0.0001* | | <0.0001* | | <0.0001 * | | <0.0001 * | |
| Pi | 0.0031 * | | 0.1474 ns | | 0.0039 * | | 0.0203 * | |
| Phi | 0.2485 ns | | 0.5378 ns | | 0.4965 ns | | 0.8070 ns | |
| PAR × Pi | 0.0807 ns | | 0.6701 ns | | 0.4522 ns | | 0.8338 ns | |
| PAR × Phi | 0.0673 ns | | 0.3851 ns | | 0.4367 ns | | 0.3942 ns | |
| Pi × Phi | 0.1157 ns | | 0.3289 ns | | 0.1370 ns | | 0.1891 ns | |
| PAR × Pi × Phi | 0.3528 ns | | 0.7803 ns | | 0.3185 ns | | 0.5935 ns | |
|  | ***Brassica juncea*** | | | | | | | |
| **Study factors and their interactions** | **Quercetin** | **Kaempferol** | | **Isorhamnetin** | | **Cyanidin** | | **Total flavonoids** |
| PAR | 0.0036 * | <0.0001 * | | 0.0017 * | | 0.0831ns | | 0.0003* |
| Pi | 0.0039 * | 0.3261 ns | | 0.0831 ns | | 0.0058 * | | 0.2521 ns |
| Phi | 0.4901 ns | 0.6969 ns | | 0.8456 ns | | 0.1364ns | | 0.7711 ns |
| PAR × Pi | 0.9017 ns | 0.2315 ns | | 0.6456 ns | | 0.3649ns | | 0.4486 ns |
| PAR × Phi | 0.0014 * | 0.0200 * | | 0.0256 * | | 0.0034 * | | 0.0019* |
| Pi × Phi | 0.4189 ns | 0.3609 ns | | 0.9732 ns | | 0.3309ns | | 0.5696 ns |
| PAR × Pi × Phi | 0.1324 ns | 0.8617 ns | | 0.6880 ns | | 0.1168ns | | 0.5102 ns |
