# Supplementary material for: Flavonoid, Nitrate and Glucosinolate Concentrations in Brassica Species Are Differentially Affected by Photosynthetically Active Radiation, Phosphate and Phosphite
Source: Front Plant Sci. 2019 Mar 27;10:371. doi: 10.3389/fpls.2019.00371 (PMC6445887; doi:10.3389/fpls.2019.00371)
Supplement: Supplementary file 5 [file Table_5.DOCX]

**Supplementary Material S5.** Statistical significance (*P*) of mean daily photosynthetically active radiation (PAR), phosphate (Pi), phosphite (Phi), and their interactions on nitrate concentrations in two *Brassica* species. Tukey’s test, ns= not significant and * significant at *P* ≤ 0.05.

| **Study factors and interactions** | ***Brassica campestris*** | ***Brassica juncea*** |
| --- | --- | --- |
| PAR | <0.0001 * | <0.0001 * |
| Pi | 0.6424 ns | 0.2144 ns |
| Phi | 0.0216 * | 0.4342 ns |
| PAR × Pi | 0.0069 * | 0.7279 ns |
| PAR × Phi | 0.1880 ns | 0.2528 ns |
| Pi × Phi | 0.3346 ns | 0.0516 ns |
| PAR × Pi × Phi | 0.0832 ns | 0.3577 ns |
