# Supplementary material for: Flavonoid, Nitrate and Glucosinolate Concentrations in Brassica Species Are Differentially Affected by Photosynthetically Active Radiation, Phosphate and Phosphite
Source: Front Plant Sci. 2019 Mar 27;10:371. doi: 10.3389/fpls.2019.00371 (PMC6445887; doi:10.3389/fpls.2019.00371)
Supplement: Supplementary file 6 [file Table_6.DOCX]

**Supplementary Material S6**. Statistical significance (*P*) of mean daily photosynthetically active radiation (PAR), phosphate (Pi), phosphite (Phi), and their interactions on glucosinolate (GL) concentration. Tukey’s test, ns= not significant and * significant at *P* ≤ 0.05.

**a)** In *Brassica* *campestris* cv. Mibuna Early.

| **Study factors**  **and interactions** | | **Alkyl-GLs** | | | | | |  | | **Alkenyl-GLs** | | | | | | | |
| --- | --- | --- | --- | --- | --- | --- | --- | --- | --- | --- | --- | --- | --- | --- | --- | --- | --- |
|  |  | **4MSB** | | | **5MSP** | **Total** | |  | | **2OH-Butenyl** | | **Butenyl** | | **Pentenyl** | | **Total** | |
| PAR | | 0.0860 ns | | | <0.0001 * | 0.0006 * | |  |  | <0.0001 * | | <0.0001 * | | <0.0001 * | | <0.0001 * | |
| Pi | | <0.0001 * | | | <0.0001 * | <0.0001 * | |  | | 0.4248 ns | | 0.2427 ns | | 0.0562 ns | | 0.7272 ns | |
| Phi | | 0.0194 * | | | 0.0008 * | 0.0009 * | |  | | 0.0003 * | | 0.8136 ns | | 0.4505 ns | | 0.7158 ns | |
| PAR × Pi | | 0.1545 ns | | | 0.2639 ns | 0.1184 ns | |  | | 0.0101 * | | 0.1297 ns | | 0.1409 ns | | 0.3583 ns | |
| PAR × Phi | | 0.4963 ns | | | 0.0380 * | 0.0944 ns | |  | | 0.0003 * | | 0.0376 * | | 0.1777 ns | | 0.0416 * | |
| Pi × Phi | | 0.0062 * | | | 0.0619 ns | 0.0080 * | |  | | 0.0846 ns | | 0.5709 ns | | 0.7509 ns | | 0.7307 ns | |
| PAR × Pi × Phi | | 0.4160 ns | | | 0.1063 ns | 0.2149 ns | |  | | 0.0951 ns | | 0.7041 ns | | 0.2892 ns | | 0.7895 ns | |
| **Study factors**  **and interactions** | **Aryl-GLs** | |  | **Indole-GLs** | | | | | | | | | | |  | **Total GLs** |  |
|  | **2PE** | |  | **1MOI3M** | | | **4MOI3M** | | **I3M** | | **4OHI3M** | | **Total** | |  |  |  |
| PAR | 0.0018 * | |  | <0.0001 * | | | 0.0799 ns | | <0.0001 * | | 0.0050 * | | <0.0001 * | |  | <0.0001 * |  |
| Pi | 0.5477 ns | |  | 0.0221 * | | | 0.0199 * | | 0.1782 ns | | 0.2474 ns | | 0.3086 ns | |  | 0.4089 ns |  |
| Phi | 0.4088 ns | |  | 0.0218 * | | | 0.0428 * | | 0.1224 ns | | 0.7914 ns | | 0.1404 ns | |  | 0.6115 ns |  |
| PAR × Pi | 0.2811 ns | |  | 0.2846 ns | | | 0.0001 * | | 0.1033 ns | | 0.6933 ns | | 0.0206 * | |  | 0.2588 ns |  |
| PAR × Phi | 0.3694 ns | |  | 0.0584 ns | | | 0.0515 ns | | 0.0876 ns | | 0.4673 ns | | 0.1413 ns | |  | 0.0340 * |  |
| Pi × Phi | 0.2835 ns | |  | 0.2346 ns | | | 0.9667 ns | | 0.1368 ns | | 0.7866 ns | | 0.4919 ns | |  | 0.7215 ns |  |
| PAR × Pi × Phi | 0.4864 ns | |  | 0.1626 ns | | | 0.3218 ns | | 0.1756 ns | | 0.2415 ns | | 0.4511 ns | |  | 0.8229 ns |  |

4MSB=4-methylsulfinylbutyl-GL; 5MSP=5-methylsulfinylpentyl-GL; 2OH-Butenyl=2-hydroxybut-3-enyl-GL; Butenyl=but-3-enyl-GL; Pentenyl=pent-4-enyl-GL; 2PE=2-phenylethyl-GL; 1MOI3M=1-methoxyindol-3-ylmethyl-GL; 4MOI3M=4-methoxyindol-3-ylmethyl-GL; I3M= indol-3-ylmethyl-GL; 4OHI3M=4-hydroxyindol-3-ylmethyl-GL.

**b)** In *Brassica juncea* cv. Red Giant.

| **Study factors**  **and interactions** | **Alkenyl-GLs** | | | | | | | | |  | | **Aryl-GLs** |
| --- | --- | --- | --- | --- | --- | --- | --- | --- | --- | --- | --- | --- |
|  | **Propenyl** | | | **Butenyl** | | **Pentenyl** | | **Total** | |  | | **2PE** |
| PAR | <0.0001 * | | | <0.0001 * | | 0.5820 ns | | <0.0001 * | |  |  | <0.0001 * |
| Pi | 0.0361 * | | | 0.6698 ns | | 0.4746 ns | | 0.0436 * | |  | | 0.0008 * |
| Phi | 0.0157 * | | | 0.0230 * | | 0.2283 ns | | 0.0158 * | |  | | 0.1907 ns |
| PAR × Pi | 0.8858 ns | | | 0.8811 ns | | 0.1606 ns | | 0.8818 ns | |  | | 0.1311 ns |
| PAR × Phi | 0.0284 * | | | 0.1378 ns | | 0.5548 ns | | 0.0301 * | |  | | <.0001 * |
| Pi × Phi | 0.5799 ns | | | 0.9551 ns | | 0.8261 ns | | 0.6070 ns | |  | | 0.0362 * |
| PAR × Pi × Phi | 0.1155 ns | | | 0.2133 ns | | 0.2937 ns | | 0.1194 ns | |  | | 0.0033 * |
| **Study factors**  **and interactions** | | **Indole-GLs** | | | | | | | |  | **Total-GLs** | |
|  |  | **1MOI3M** | **4MOI3M** | | **I3M** | | **4OHI3M** | | **Total** |  |  |  |
| PAR | | <0.0001 * | 0.3007 ns | | <0.0001 * | | <0.0001 * | | <0.0001 * |  | <0.0001 * | |
| Pi | | 0.8326 ns | 0.9105 ns | | 0.3548 ns | | 0.0385 * | | 0.1798 ns |  | 0.0425 * | |
| Phi | | 0.5237 ns | 0.2062 ns | | 0.3535 ns | | 0.0002 * | | 0.0475 * |  | 0.0160 * | |
| PAR × Pi | | 0.2061 ns | 0.2379 ns | | 0.1031 ns | | 0.0109 * | | 0.5005 ns |  | 0.8601 ns | |
| PAR × Phi | | 0.2605 ns | 0.1876 ns | | 0.0023 * | | 0.0493 * | | 0.0935 ns |  | 0.0287 * | |
| Pi × Phi | | 0.6482 ns | 0.8784 ns | | 0.0275 * | | 0.2172 ns | | 0.7784 ns |  | 0.6186 ns | |
| PAR × Pi × Phi | | 0.6471 ns | 0.7304 ns | | 0.0321 * | | 0.0014 * | | 0.0184 * |  | 0.1080 * | |

Propenyl=prop-2-enyl-GL; Butenyl=but-3-enyl-GL; Pentenyl=pent-4-enyl-GL; 2PE=2-phenylethyl-GL; 1MOI3M=1-methoxyindol-3-ylmethyl-GL; 4MOI3M=4-methoxyindol-3-ylmethyl-GL; I3M=indol-3-ylmethyl-GL; 4OHI3M=4-hydroxyindol-3-ylmethyl-GL.
